# Citric Acid Promoting B Lymphocyte Differentiation and Anti-epithelial Cells Apoptosis Mediate the Protective Effects of *Hermetia illucens* Feed in ETEC Induced Piglets Diarrhea

**DOI:** 10.3389/fvets.2021.751861

**Published:** 2021-11-30

**Authors:** Mingming Liu, Boyu Yuan, Xinxin Jin, Mingqiang Zhu, Haidong Xu, Gaijie Xie, Zifan Wang, Xue Zhang, Zhaoyun Xu, Bai Li, Yanhua Huang, Yantao Lv, Wei Wang

**Affiliations:** ^1^College of Animal Science & Technology, Innovative Institute of Animal Healthy Breeding, Zhongkai University of Agriculture and Engineering, Guangzhou, China; ^2^College of Veterinary Medicine, Jilin University, Changchun, China; ^3^State Key Laboratory of Oncology in South China, Collaborative Innovation Center for Cancer Medicine, Sun Yat-sen University Cancer Center, Guangzhou, China; ^4^Special Police Academy of Chinese People's Armed Police Force, Beijing, China; ^5^The First Hospital of Jilin University, Changchun, China

**Keywords:** weaning piglets, enterotoxigenic *Escherichia coli* (ETEC) K88, *Hermetia illucens*, citric acid (CA), B lymphocyte, intestinal barrier

## Abstract

Newborn piglets are prone to diarrhea after weaning as a result of changes in their environment and feed. Enterotoxigenic *Escherichia coli* (ETEC) K88 strain is a typical pathogen that causes diarrhea in such stage of piglets. *Hermetia illucens* larvae are widely used in livestock and poultry production because of their high nutritional value and immunoregulatory effects. This study aimed to evaluate the protective effects of *H. illucens* feed in protecting against ETEC induced diarrhea in piglets and to unravel the mechanisms of immune modulation and intestinal barrier maintenance. The results showed that after ETEC infection, citric acid in the serum of the groups fed on *H. illucens* larvae increased significantly, which stimulated macrophages to secrete cytokines that promote B lymphocyte differentiation, ultimately increasing the production of IgA and IgG in serum. Concomitantly, citric acid also had a positive effect on the intestinal barrier damaged due to ETEC infection by inhibiting the production of inflammatory cytokines, reducing the Bcl-2/Bax ratio, and promoting the expression of tight junction proteins. Correlation analysis showed that the increase of citric acid levels might be related to *Massilia*. Thus, citric acid derived from *H. illucens* larvae can improve the immune performance of weaned piglets and reduce ETEC-induced damage to the intestinal barrier in weaned piglets.

## Introduction

After the early weaning of newborn piglets, the intestinal flora is not yet well-established, and the intestinal barrier is not yet mature. Due to environmental changes, feed stress, and other risk factors, newborn piglets are often easily infected by pathogenic bacteria, mainly the enterotoxigenic ETEC K88, to develop diarrhea (1). ETEC K88 colonizes on the surface of the host's gastrointestinal tract and produces enterotoxins together with lipopolysaccharides to induce an abnormal immune response, destroy the electrolyte homeostasis of intestinal cells and ultimately lead to secretory diarrhea in piglets (2). In China, the mortality rate of post-weaning diarrhea (PWD) due to ETEC K88 infection is relatively high, causing serious losses to the breeding industry (3). Antibiotics have been widely used in the breeding industry for more than 50 years because they can effectively control bacterial diseases (4). However, the massive use of antibiotics leads to the development and spread of antibiotic-resistant bacteria (5, 6). Following the European Union, in 2020, China banned the use of antibiotics in pig feed. Therefore, finding a new and effective antibiotic substitute for pigs has become necessary—especially for weaned piglets because of their poor immune system and weaning stress—to treat and prevent bacterial infections, including diarrhea caused by ETEC K88.

In the animal breeding industry, the use of insects as alternative feed ingredients has become a consensus, not only because of the significant nutritional properties and favorable feeding characteristics (7) but also because of the ability to regulate the intestinal microbiota and positively impact animal health (8). Among the insect species studied for animal feeding purposes, *H. illucens* has recently gained the most attention in the pig rearing industry (9, 10). The larvae of *H. illucens* are rich in nutrition, antimicrobial peptides, and chitin that have probiotic effects on the host and have immune regulatory effects on the host. It has been used as high-quality feed material in livestock and poultry production. *H. illucens* prepupa and larval meals are highly digestible and safe for weaning piglets, and no negative effects on animal health and production performance and intestinal mucosal morphology have been observed (11, 12). Recently, *H. illucens* larvae meals were reported to enhance the colonic mucosal immune homeostasis of fattening pigs by actively changing the bacterial composition and its metabolites (13). However, only a few studies have been reported on the protective mechanism of *H. illucens* larvae on the weaning process of piglets, especially on weaning piglets under the invasion of pathogenic bacteria.

Based on the above-mentioned reports, ETEC K88 was used to establish a diarrhea model for weaned piglets, and the protective effect and the role of *H. illucens* larvae feed in ETEC K88 infected piglets were explored. *H. illucens* larvae were found to improve the immune performance and repair intestinal barrier damage caused by the ETEC K88 and thus provided new ideas for the treatment of diseases caused by other pathogenic microorganisms.

## Materials and Methods

### Preparation of *H. illucens* Feed

The piglet feed was supplemented with 0, 4, and 8% black soldier fly insect powder (C group, T1 group, T2 group). The feed for all groups was prepared in accordance with NRC (2012) standards.

### Handling of Weaned Piglets

For this study, 12 weaned piglets with an initial average weight of 7.68 ± 0.26 kg were randomly categorized into C group (K88 group), T1 group (4% *H. illucens* larvae + K88 group), and T2 group (8% *H. illucens* larvae + K88 group) with four replicates in each group. The feed for all groups was prepared in accordance with NRC (2012) standards. Piglets were fed on a diet supplemented with 0, 4, and 8% black soldier fly insect powder (C group, T1 group, T2 group) for 28 days. On the 29th day, the ETEC K88 (50 × 10^9^ CFU/mL) was infused in the piglets using an intragastric catheter (14, 15). On the 32nd day, 10 mL of blood from the anterior vena cava of the piglets was collected in tubes containing an anticoagulant and used for metabolite detection. Ileum tissue was taken and placed in PBS buffer to wash away the contents, divided into two portions. One portion was stored in 4% formaldehyde solution for sectioning, and from the other portion, the intestinal mucosa was scraped for detecting related protein expression.

### 16S rRNA Sequence

For examining the microbial diversity, the Illumina HiSeq sequencing platform was used to construct a small fragment library and sequenced, followed by diversity analysis, difference analysis, correlation analysis, and function prediction analysis as previously described (16).

### Gas Chromatography-Mass Spectrometry

To Detect the Effect of *H. illucens* on Metabolites in Serum of Weaned Piglets, GC-MS was performed to detect metabolites such as organic acids, fatty acids, and indole in serum (17, 18).

### Mice Treatment

In this study, male mice born in the same litter and aged 6–8 weeks were used after procuring them from Zhuhai Bestone Biotechnology Co., Ltd. The mice were kept in an environment with freely available feed, natural light, and temperature at 22–25°C. To establish an ETEC infection model, Allen's method (19) was referred. In brief, water (5 g/L) supplemented with streptomycin was given to all mice for 72 h before ETEC infection to clear the natural flora in the intestines. Twelve hours before ETEC inoculation, the streptomycin-treated water was replaced with sterile water, and 1 × 10^9^ CFU of ETEC strain K88 was diluted with 0.1 M carbonate buffer, pH 9.0, and given to mice by gavage (20). The control group was given an equal volume of sterile phosphate-buffered saline (PBS). Two weeks before drinking streptomycin, the mice were given citric acid (5 g/L) by adding to the drinking water until the end of the experiment (21). Five days after ETEC infection, the animals were sacrificed to collect serum, spleen, and ileum and stored in a refrigerator at −80°C.

### Cell Culture

IPI-2I cells and RAW264.7 macrophages, purchased from BeiNa Culture Collection (Beijing, China), were cultured in Dulbecco's modified Eagle's medium (Gibco, Grand Island, NY) containing 10% fetal bovine serum (Clark, Australia) and were maintained at 37°C in a humidified chamber of 5% CO_2_.

### *In vitro* Experiments

In this study, RAW264.7 cells were categorized into four groups. The control group was cultured with D-MEM/F-12 containing 10% fetal bovine serum. The LPS group was treated with 1 μg/mL LPS for 24 h. The CA group was treated with 4 mmol/L citric acid for 24 h. The CA pretreatment group (CA+LPS group) was pre-protected with 4 mmol/L citric acid for 1 h and then treated with 1 μg/mL LPS for 24 h. The treatment of IPI-2I cells was consistent with that of RAW264.7 cells. The Cell Counting Kit-8 assay estimated the optimal concentration of lipopolysaccharides (LPS) and CA to be 1 μg/mL and 4 mmol/L, respectively.

### RNA Extraction and Real-Time Reverse Transcription-Polymerase Chain Reaction

Total RNA was extracted from the ileum tissue, RAW264.7 macrophages, primary splenic cells, and IPI-2I cells using TRIzol (Invitrogen, Carlsbad, CA), and cDNA was generated using a commercial RT-PCR kit (Takara Shuzo Co., Ltd., Kyoto, Japan). Then, real-time PCR was conducted using the SYBR Green QuantiTect RT-PCR kit (Roche, South San Francisco, CA), and each sample was analyzed in triplicate. The primer sequences for mice and Sus scrofa are shown in Tables 1, 2.

**Table 1:** Primers for Real-time PCR (mice).

**Gene**	**Sequences**	**Length (bp)**
IL-1β	Forward: 5′- TGTGATGTTCCCATTAGAC−3′	87
	Reverse: 5′- AATACCACTTGTTGGCTTA−3′	
IL-6	Forward: 5′- AGCCACTGCCTTCCCTAC−3′	133
	Reverse: 5′- TTGCCATTGCACAACTCTT−3′	
TNF-α	Forward: 5′- CCACGCTCTTCTGTCTACTG−3′	110
	Reverse: 5′- GCTACGGGCTTGTCACTC−3′	
Bax	Forward: 5′-AAACTGGTGCTCAAGGCCCT-3′	92
	Reverse: 5′-AGCAGCCGCTCACGGAG-3′	
Bcl-2	Forward: 5′-AGCGACGAGAGAAGTCATCC-3′	
	Reverse: 5′-CTGTAGCATGGGCATCCTTT-3′	183
Cldn5	Forward: 5′- GTGGCACTCTTTGTTACCTTG-3′	172
	Reverse: 5′- GATCATAGAACTCGCGGACAA-3′	
IL-10	Forward: 5′- TTCTTTCAAACAAAGGACCAGC−3′	81
	Reverse: 5′- GCAACCCAAGTAACCCTTAAAG-3′	
BAFF	Forward: 5′- GGACACTGGACATACAAGCAG−3′	211
	Reverse: 5′- GACTCCTTCTGAAGTGTCTGG-3′	
TGF-β	Forward: 5′- ATTGCTGCCTTCGCCCTCTTTAC-3′	150
	Reverse: 5′-GGCTGAGGACTTTGGTGTGTTGAG-3′	
Occludin	Forward: 5′- ACACTTGCTTGGGACAGAGG−3′	197
	Reverse: 5′- AAGGAAGCGATGAAGCAGAA−3′	
GAPDH	Forward: 5′-GCCATCACTGCCACCCAGAA-3′	153
	Reverse: 5′-GCCAGTGAGCTTCCCGTTGA-3′	

**Table 2:** Primers for real-time PCR (Sus scrofa).

**Gene**	**Sequences**	**Length (bp)**
IL-1β	Forward:5′-TGGTGTCTGTGATTGTGGCAAAGG-3′	120
	Reverse:5′-TTTCAAGGACGATGGGCTCTTCTTC−3′	
IL-6	Forward:5′- ATAAGGGAAATGTCGAGGCTGTGC−3′	93
	Reverse:5′- GGGTGGTGGCTTTGTCTGGATTC-3′	
TNF-α	Forward:5′- GCCTCTTCTCCTTCCTCCTG−3′	193
	Reverse:5′- TCGGCTTTGACATTGGCTAC−3′	
Bax	Forward:5′-GCTTCAGGGTTTCATCCAGGATCG-3′	107
	Reverse: 5′-ACTCGCTCAACTTCTTGGTAGATGC-3′	
Bcl-2	Forward:5′-TCGCCCTGTGGATGACTGAGTAC-3′
	Reverse:5′-CCTTCAGAGACAGCCAGGAGAAATC-3′	138
Occludin	Forward: 5′-TGGCTGCCTTCTGCTTCATTGC -3′	
	Reverse: 5′-GAACACCATCACACCCAGGATAG -3′	131
ZO-1	Forward: 5′-GCCATCCACTCCTGCCTAT -3′	
	Reverse: 5′-CGGGACCTGCTCATAACTTC -3′	133
GAPDH	Forward: 5′-GCCATCACTGCCACCCAGAA−3′	153
	Reverse: 5′-GCCAGTGAGCTTCCCGTTGA-3′	

### Western Blotting

Total proteins were extracted from the ileum tissue and IPI-2I cells following the procedure described previously (22). Subsequently, western blotting was performed following the standard protocol (22). The antibodies used were: Occludin (1:1,000; EPR8208, Abcam), Bax (1:5,000; 50599-2-lg, Proteintech), Bcl-2 (1:1,000; 12789–1-AP, Proteintech), β-Actin (1:1,000; 4970, CST), HRP Conjugated AffiniPure Goat Anti-Rabbit IgG (1:5,000; BA1055, BOSTER).

### Enzyme-Linked Immunosorbent Assay

Elisa kits (MEIMIAN, MM-0057M1, MM-0055M1, MM-45051M1, and MM-44850M1) were used. Detection of IgA and IgG in piglet serum, IgA, IgG, B cell-activating factor (BAFF), TGF-β, and IL-10 in mouse serum and BAFF, TGF-β, and IL-10 in RAW264.7 cell culture medium. Specific steps were performed following instructions from the manufacturer.

### Flow Cytometry Analysis

Mouse primary splenocyte suspension was collected and stained with combinations of the following antibodies: BV421 anti-IgG, AF750 anti-CD19 (Biolegend), PE anti-CD138 (Syndecan-1, R&D Systems), and FITC anti-IgA (Southern Biotech). For intracellular detection of IgG and IgA, Golgi-stop was added to cell cultures 4 h prior to harvest and extracellular staining was performed using anti-CD19 and anti-CD138 Abs. Cells were then fixed and permeabilized before intracellular staining with anti-IgG and anti-IgA Abs.Stained cells were analyzed on an Attune NxT flow cytometer (Thermo Fisher Scientific, Waltham, MA).

### HE and TUNEL Staining

The ileum tissues of piglets and mice were taken, and conventional slices were prepared. One was used for HE staining to observe the pathological changes of the tissue under a light microscope, and the other was used for TUNEL staining to observe the apoptosis ratio in the tissues, strictly following the kit instructions.

### Cell Counting Kit-8 Assay

RAW264.7 cells were centrifuged and seeded in 96-well plates at a density of 2 × 10^4^ cells/well, with five repetitions for each treatment group. Twenty-four hours after seeding the cells, the medium was changed to DMEM basal medium. After 4 h of treatment, citric acid was added for pre-protection, and then 2 h later LPS was added for stimulation. After 24 h of stimulation, the medium was discarded, and 100 μL of CCK8 premix solution was added to each well. Finally, after 1 h, the absorbance was detected at 460 nm.

### Statistical Analysis

The results are expressed as mean ± SEM. Statistical analysis was performed using Student's *t*-test or one-way analysis of variance (ANOVA) and analyzed using GraphPad Prism 7.0 (GraphPad Software, San Diego, CA). The *P* value < 0.05 was considered statistically significant.

## Results

### *H. illucens* Larvae Feed Improved the Immune Performance and Repaired Intestinal Barrier Damage Caused by ETEC K88 Infection

**Figure 1:**
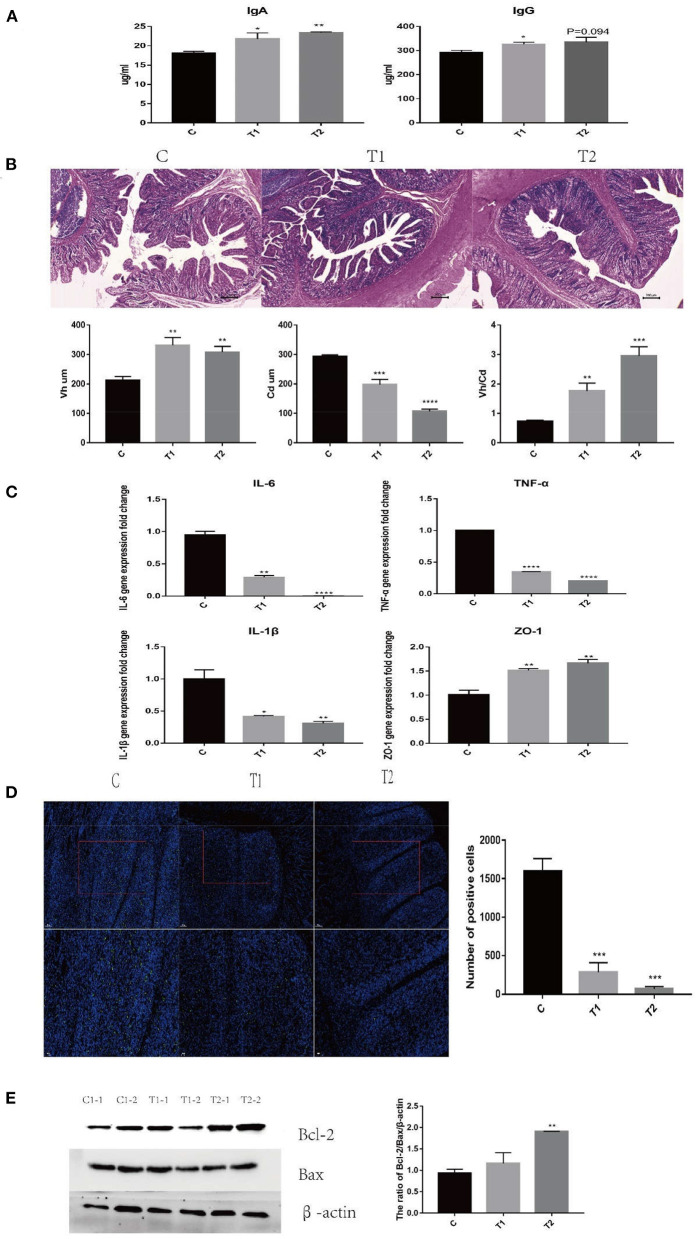
*Hermetia illucens* larval feed improves the immune performance of piglets and repairs the intestinal barrier damage caused by ETEC K88 infection. **(A)** IgA and IgG secreted in piglet serum were measured by ELISA (*n* = 4). **(B)** The ratio of Vh/Cd (villus height/crypt depth) of piglet ileum was immunohistochemically analyzed. **(C)** The mRNA expression of TNF-α, IL-6, Zo-1, and IL-1β was assessed by RT-PCR. **(D)** TUNEL staining analysis of ileum cell apoptosis in piglets. **(E)** Effects of *H. illucens* larval feed on ileum anti-apoptotic ratio (*n* = 4, means ± SEM, ANOVA, * *P* < 0.05, ** *P* < 0.01, *** *P* < 0.001, **** *P* < 0.0001).

After ETEC infection, the serum IgA and IgG levels of piglets in the *H. illucens* larvae feed groups were significantly higher than those in the C group (Figure 1A). HE staining results showed that the ileum Vh/Cd (villus height/crypt depth) ratio of the *H. illucens* larvae feed groups was significantly higher than that of the C group (Figure 1B). The gene expression of the inflammatory factors IL-6, TNF-α, IL-1β, and tight junction protein ZO-1 in the ileal mucosa was also detected (Figure 1C). The expression of TNF-α and IL-1β in the *H. illucens* larvae feed groups decreased significantly, but the expression of ZO-1 increased significantly. Concomitantly, the results of TUNEL staining showed that compared with the C group, ETEC K88-mediated apoptosis was significantly inhibited in the *H. illucens* larvae feed groups (Figure 1D). The changes in the ratio of Bcl-2/Bax were detected by western blotting, and the results reveal significantly increased anti-apoptotic Bcl-2/Bax ratio of the *H. illucens* larvae feed groups (Figure 1E). The above results indicate that under the challenge of ETEC K88, *H. illucens* larvae feed not only improved the immune performance but also attenuated the damage caused by ETEC K88 by reducing apoptosis and inflammation, and enhancing the integrity of the intestinal barrier.

### Citric Acid Levels Increased in Serum Samples With the Addition of *H. illucens* Larvae

**Figure 2:**
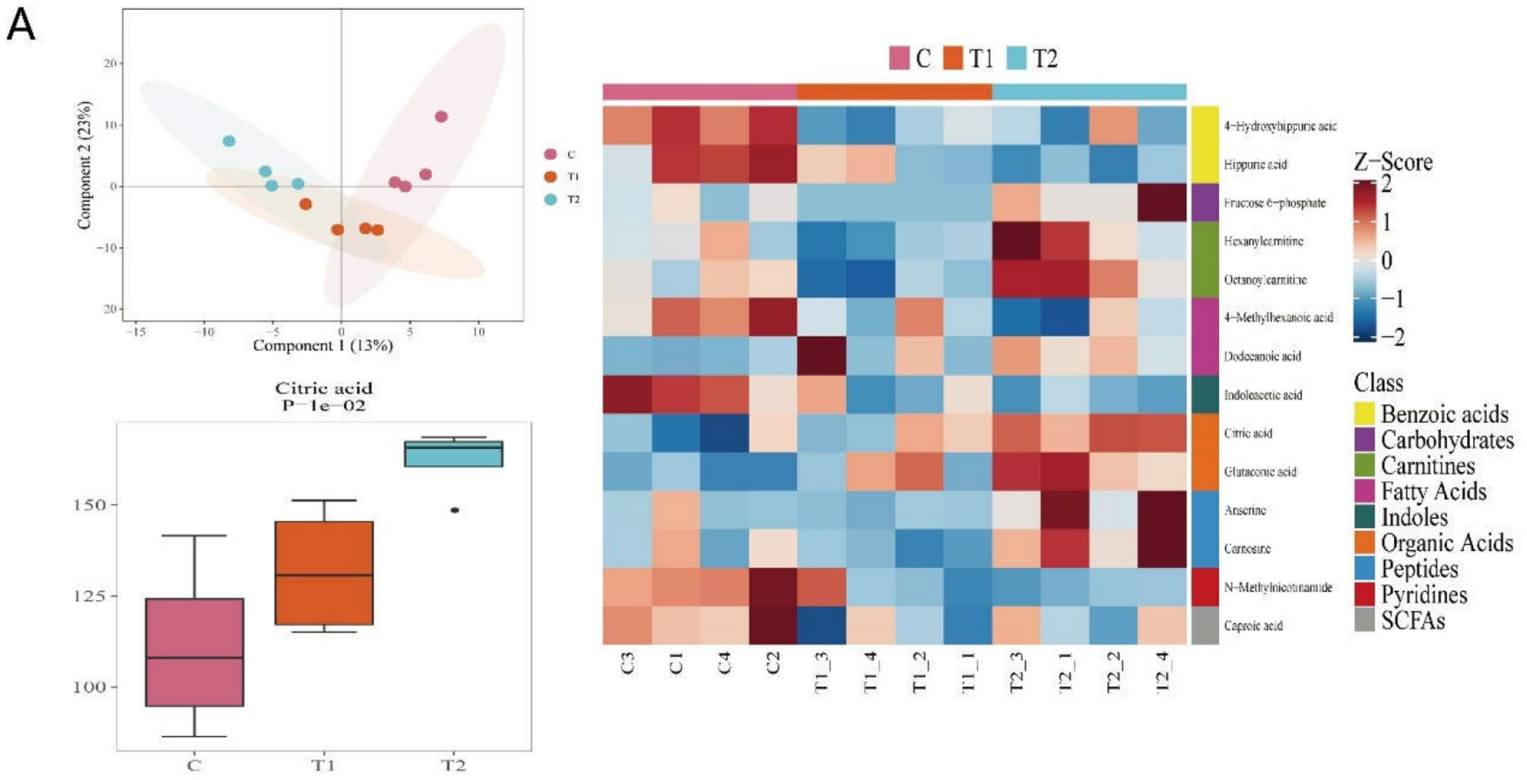
The feed of *Hermetia illucens* larvae increased the content of metabolite citric acid (CA) in piglet serum samples. The results of **(A)** serum metabolomics test (*n* = 4, means ± SEM).

In order to reveal the specific mechanism in piglets under *H. illucens larvae* supplement, metabonomic analyses were carried out on the serum of the three groups of piglets. We found that with the increase in the content of *H. illucens* larvae, the citric acid content in the serum (Figure 2A), which was confirmed to be related to inflammation and immunity, increased in a dose-dependent manner. Therefore, we suppose that *H. illucens* larvae might affect the immune performance and intestinal health of piglets through citric acid derived from it.

### Citric Acid Inhibits Excess Production of Inflammatory Cytokines in RAW264.7 Cells

**Figure 3:**
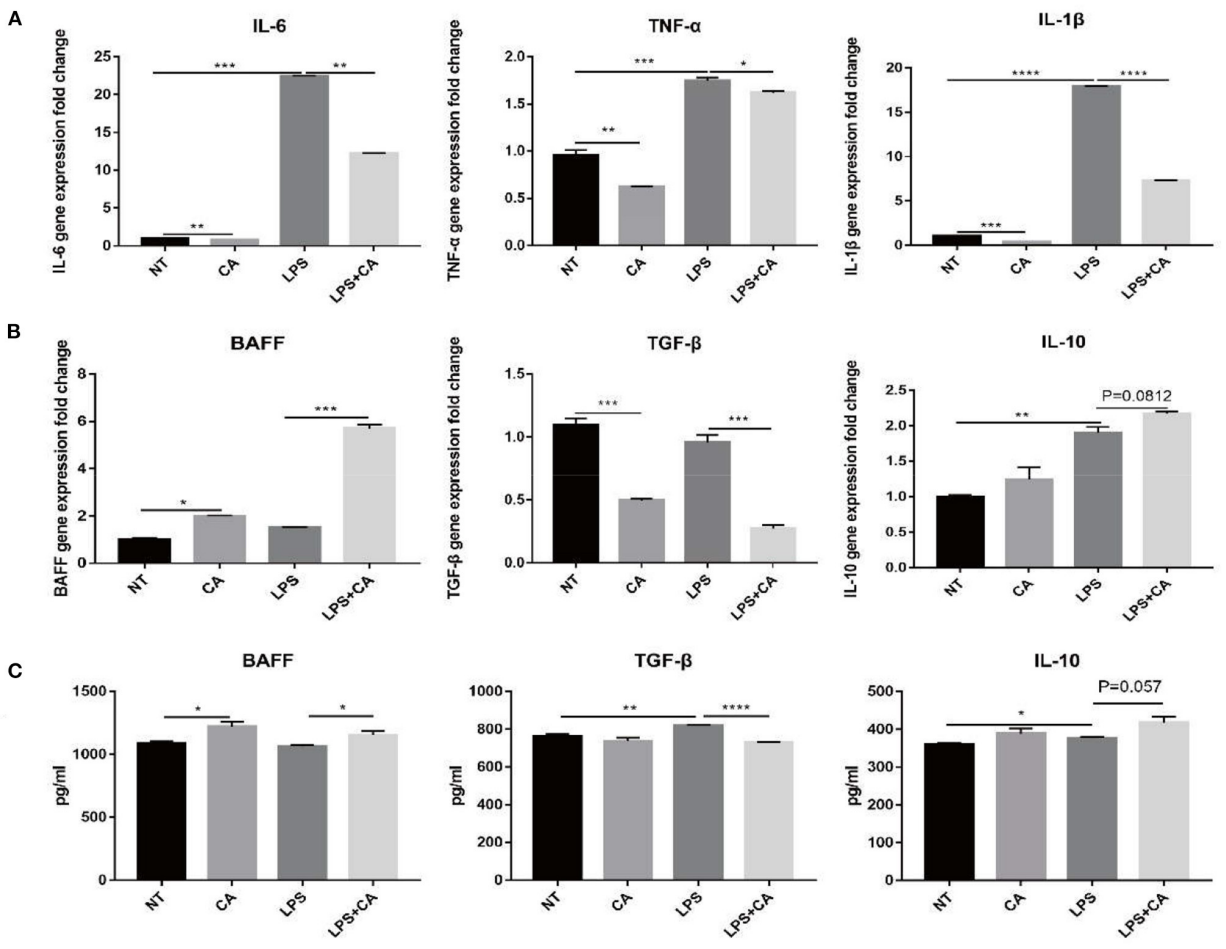
Citric acid (CA) inhibited the overproduction of inflammatory factors in RAW264.7 cells and promoted the production of cytokines related to B lymphocyte activation. After treatment with 4 mmol/L citric acid for 2 h, LPS (1 μg/mL) was added and after 24 h, the sample was collected for total RNA was extraction. **(A)** Expression of IL-6, TNF-α, and IL-1β was detected by RT-PCR. **(B)** Expression of BAFF, TGF-β and IL-10 was detected by RT-PCR. **(C)** Detection of BAFF, TGF-β and IL-10 secreted in supernatants of RAW264.7 cells by ELISA (*n* = 3, means ± SEM, Student's *t*-test, * *P* < 0.05, ** *P* < 0.01, *** *P* < 0.001, **** *P* < 0.0001).

In order to confirm the relationship between the CA derived from *H. illucens* larvae and the immune performance of piglets, mouse peritoneal macrophages RAW264.7 cells were used to study the immune regulation mechanism of citric acid *in vitro*. On analyzing gene expression of non-specific immune-related cytokines IL-6, TNF-α, and IL-1β, we observed a significant increase in the expression of non-specific immune-related cytokines IL-6, TNF-α, and IL-1β after LPS challenge (Figure 3A). Compared with the LPS group, the LPS+CA group exhibited a significantly attenuated increase in the expression of IL-6, TNF-α, and IL-1β (Figure 3A). The above results showed that the treatment of citric acid can significantly inhibit the excessive production of inflammatory factors IL-6, TNF-α, and IL-1β in RAW264.7 cells induced by LPS.

### Citric Acid Promotes the Production of Cytokines Related to B Lymphocyte Differentiation in RAW264.7 Cells

The expression of specific immune-related cytokines, such as BAFF, TGF-β, and IL-10, indicated that citric acid treatment reduced the expression of BAFF significantly; however, that of TGF-β and IL-10 increased significantly (Figure 3B). In addition, compared with the NT group, the CA group exhibited significantly increased expression of BAFF and decreased expression of TGF-β, while an increase in IL-10 level was also observed (Figure 3B). Likewise, we also assessed the secretion of specific immune-related cytokines by macrophages, and the results were consistent with their mRNA levels (Figure 3C). The above results showed that in the case of LPS challenge, the addition of citric acid could promote the secretion of BAFF, a B cell-activating factor by macrophages, and concurrently reduce the release of TGF-β, which promotes the activation and proliferation of B cells. Therefore, we preliminarily speculated that CA might be related to the B lymphocyte proliferation or differentiation.

### Citric Acid Inhibited the Proliferation of B Lymphocytes in Mouse Spleen Cells

**Figure 4:**
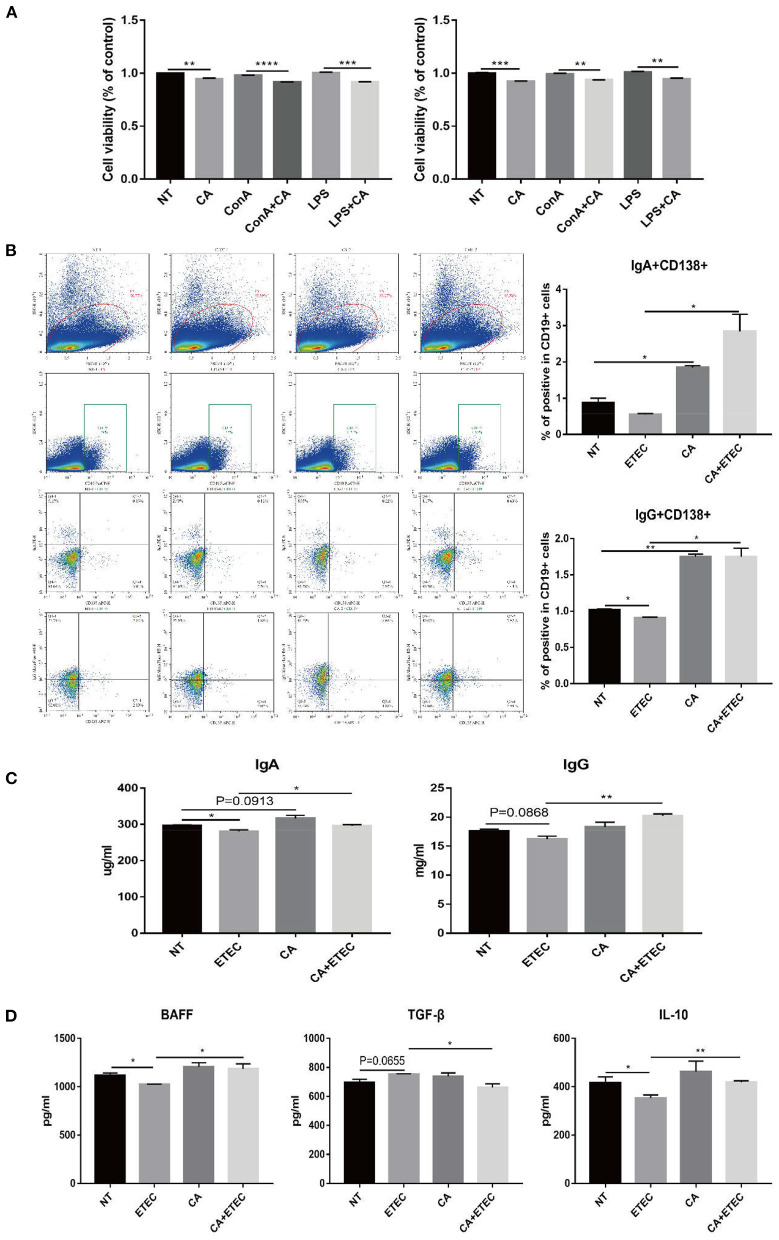
Citric acid (CA) inhibited B cell proliferation but promoted IgA+ and IgG+ B cells in mouse spleen. **(A)** The effect of CA on the proliferation of mouse splenic B cells was detected by Cell Counting Kit-8 Assay. Cells treated with 20 μg/mL ConA were used as a positive control for T cell proliferation, and 1 μg/mL LPS was used as a positive control for B cell proliferation. **(B)** Flow cytometry analysis of the effect of CA on the differentiation of mouse spleen B cells. Cells were stained extracellularly with anti-CD19 and anti-CD138 antibodies and intracellularly with anti-IgG and anti-IgA antibodies. The left side of the picture shows the gating strategy for analysis of IgG^+^CD138^+^ and IgA^+^CD138^+^ cells among CD19+ cells. The right side of the picture presents percentage of CD138^+^ IgG^+^ and CD138^+^ IgA^+^ among CD19^+^ B cells. All mice were given water containing streptomycin (5 g/L) for 72 h, then changed to water supplemented with CA (5 g/L), which was continued until the end of the experiment. Before ETEC infection, mice were starved for 12 h, and the treated mice were orally inoculated (gavage) with 1 × 10^9^ colony-forming units (CFUs) of the ETEC strain K88, diluted with 0.1 M carbonate buffer (pH 9.0). The control group was orally administered an equal volume of sterile phosphate buffer. Five days after infection, the mice were sacrificed to extract splenocytes for flow cytometry. **(C)** Levels of IgA and IgG secreted in mice serum were measured by ELISA. **(D)** Levels of BAFF, TGF-β, and IL-10 secreted in mouse serum were measured by ELISA (*n* = 3, means ± SEM, Student's *t*-test, * *P* < 0.05, ** *P* < 0.01, *** *P* < 0.001, **** *P* < 0.0001).

To further verify the relationship between CA and B cell proliferation, primary mouse spleen cells were extracted and cultured. For T lymphocyte proliferation, ConA was used as a positive control, and for B lymphocyte proliferation, LPS was used as a positive control. CCK8 assay at 24 h showed a significant decrease in cell viability in the CA group compared with that of the NT group (Figure 4A). At the same time, the cell viability of the ConA+CA group was significantly lower than that of the ConA group, and a similar trend was observed for the LPS+CA group and the LPS group (Figure 4A). The result of CCK8 at 48 h was consistent with that at 24 h, further confirming that citric acid inhibited B cell proliferation (Figure 4A).

### Citric Acid Promoted the Transformation B Lymphocytes Into Antibody-Secreting Plasma Cells and the Production of IgA and IgG in Mouse Splenocytes

To verify the effect of citric acid on B cell activation, C57BL/6 mice were categorized into NT group, ETEC group, CA group, and CA+ETEC group. Flow cytometry of primary spleen cells of mice revealed that ETEC infection significantly reduced percentage of the IgG^+^ cells among CD19^+^ B cells, and a similar trend was observed for IgA^+^ B cells (Figure 4B). Compared with the NT group, citric acid significantly increased the IgG^+^ and IgA^+^ cell ratio in CD138^+^ CD19^+^ cells (Figure 4B). At the same time, citric acid significantly reversed the decrease in IgG^+^ and IgA^+^ population in CD138^+^CD19^+^ cells caused by ETEC infection (Figure 4B). To further verify results, the levels of IgA and IgG in the serum of mice were tested, and the results were consistent with those of flow cytometry (Figure 4C). Estimation of BAFF, TGF-β, and IL-10 levels in the serum of mice showed that upon ETEC infection, the contents of BAFF in the serum of mice reduced significantly, the addition of citric acid significantly alleviates BAFF reduction compared with that in the ETEC group (Figure 4D). The level of IL-10 was consistent with that of BAFF (Figure 4D). Likewise, the TGF-β level increased significantly after ETEC infection, citric acid attenuates the increased TGF-β (Figure 4D). The above experimental results showed that citric acid could stimulate macrophages to secrete a large amount of cytokines that promoted B cell activation, thereby increasing IgA and IgG production. These results were also found consist with increased IgA and IgG levels in the serum of piglets in the *H. illucens* larvae feed group.

### Citric Acid Attenuated the Damage Caused by Lipopolysaccharides to IPI-2I Cells

**Figure 5:**
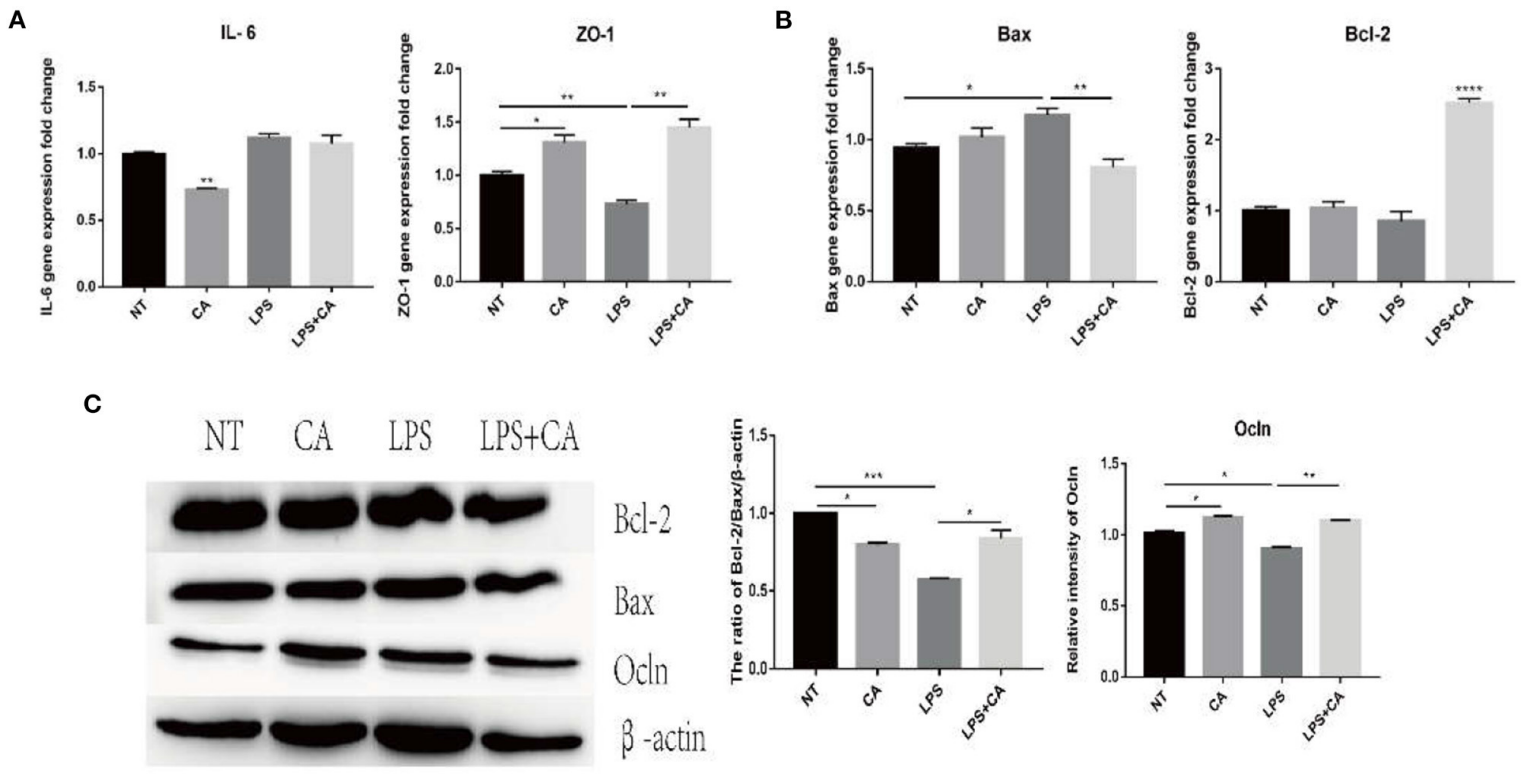
Citric acid (CA) significantly attenuated the damage caused by LPS to IPI-2I cells. After the cells were treated with 4 mmol/L CA for 2 h, LPS (1 μg/mL) was added for 24 h, and the samples were collected for total RNA and total protein extraction. **(A,B)** Expression of IL-6, ZO-1, Bax, and Bcl-2 was detected by RT-PCR. **(C)** Representative immunoblot of Ocln, Bax, and Bcl-2 expression in IPI-2I cells (*n* = 3, means ± SEM, Student's *t*-test, * *P* < 0.05, ** *P* < 0.01, *** *P* < 0.001, **** *P* < 0.0001).

To verify the effect of CA on intestinal epithelial integrity, the porcine ileal epithelial cell line IPI-2I was cultured and divided into NT, CA, LPS, and LPS+CA groups. Assessment of the expression of genes coding for inflammatory cytokine IL-6, tight junction protein ZO-1, and apoptosis-related proteins Bcl-2 and Bax, and tight junction protein Ocln showed that after LPS stimulation, the mRNA level of IL-6 increased significantly, while that of ZO-1 decreased significantly, and the level of Ocln protein decreased significantly (Figures 5A–C). Concurrently, the anti-apoptotic ratio of Bcl-2/Bax also decreased significantly (Figures 5B,C). Compared with the NT group, the CA group revealed significantly increased expression of tight junction proteins ZO-1 and Ocln, increased anti-apoptotic ratio of Bcl-2/Bax, and decreased expression of the inflammatory cytokine IL-6 (Figures 5A–C). These results indicate that citric acid inhibited the release of IL-6, promoted the expression of intestinal tight junction protein, and led to an increase in the anti-apoptotic ratio of cells, thus maintaining intestinal integrity.

### Citric Acid Inhibited the Damage to the Intestinal Tract of Mice Induced by ETEC Infection

**Figure 6:**
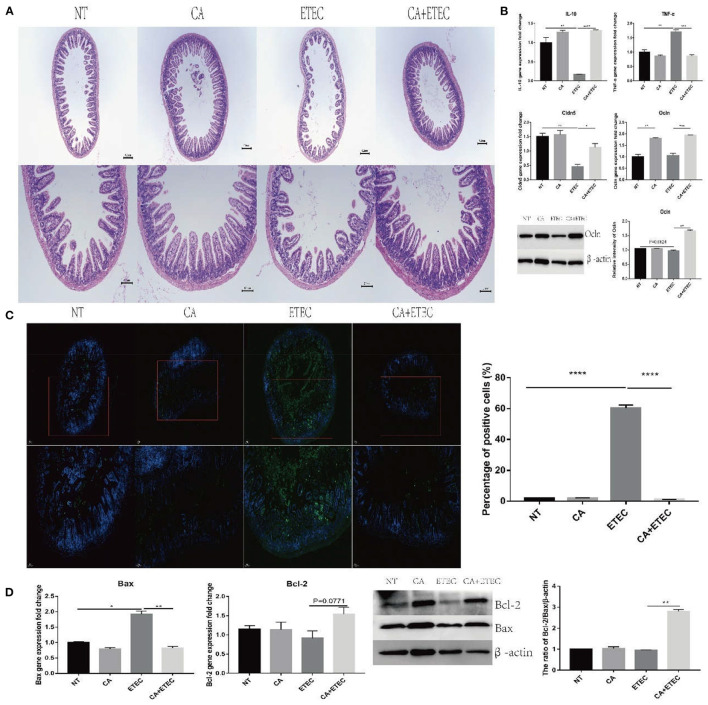
Citric acid (CA) inhibited the inflammation and apoptosis of ileum in mice infected by ETEC. **(A)** HE staining analysis of ileum of mice. **(B)** Expression of IL-10, TNF-α, Cldn5, and Ocln was detected by RT-PCR. **(C)** TUNEL staining analysis of ileum of mice. **(D)** The anti-apoptotic effects of CA on mice ileum. Representative immunoblot of Bax and Bcl-2 expression in mouse ileum (*n* = 3, means ± SEM, Student's *t*-test, * *P* < 0.05, ** *P* < 0.01, *** *P* < 0.001, **** *P* < 0.0001).

Since citric acid has a protective effect on porcine ileal epithelial cells, the protective effects of citric acid were evaluated in an ETEC infected mice model. HE staining showed that after ETEC K88 infection, the ileal villi were sparse and messy, while those in the CA group were denser and more regular (Figure 6A). Compared with the ETEC group, the addition of citric acid made the ileal villi more compact and regular (Figure 6A). At the same time, after ETEC K88 infection, the expression of TNF-α in the jejunum increased significantly, that of IL-10 decreased significantly, and the expression of Cldn5 and Ocln also decreased significantly (Figure 6B). Compared with the NT group, the CA group exhibited significantly reduced expression of inflammatory factor TNF-α and promoted the expression of the anti-inflammatory cytokine IL-10 and the tight junction proteins Cldn5 and Ocln (Figure 6B). Compared with the ETEC group, the addition of citric acid inhibited the increase in TNF-α expression and the decrease in the expression of tight junction proteins caused by ETEC infection (Figure 6B).

TUNEL results showed a significantly increased percentage of apoptotic area in the ETEC group, and there was no difference between the CA group and the NT group (Figure 6C). Compared with the ETEC group, the addition of citric acid significantly reduced the percentage of the apoptotic area (Figure 6C) and increased the anti-apoptotic ratio of Bcl-2/Bax (Figure 6D).

### The 16S Sequencing and Metabolomics Analyses Showed That *Massilia* Positively Correlated With Citric Acid in Serum

**Figure 7:**
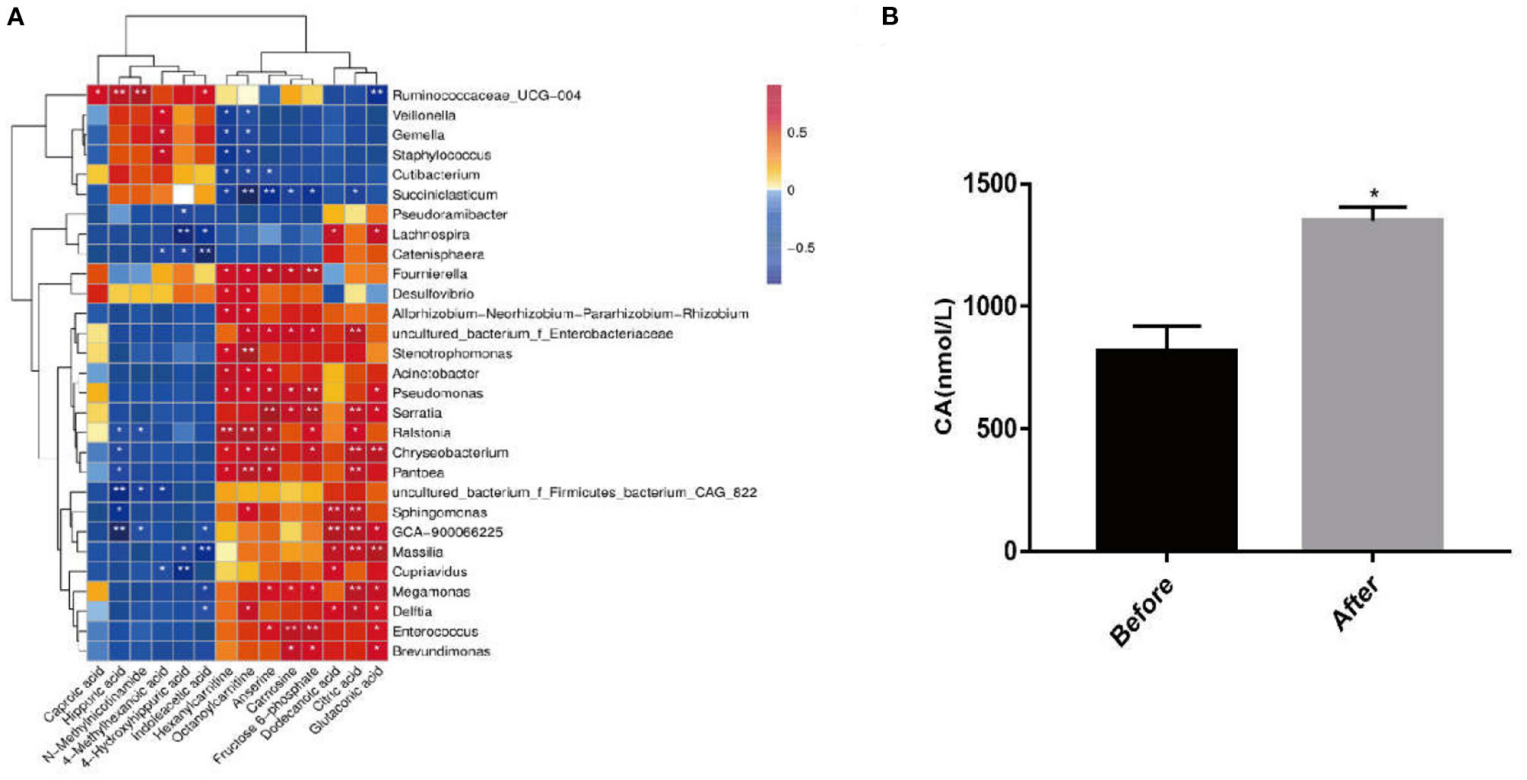
*Massilia* can produce citric acid. The samples were divided into two groups, the before group (containing only 5 mL R2A liquid medium) and the after group (5 mL R2A liquid medium with 5 μL of *Massilla, Massilla* purchased from GDMCC), placed in a shaker at 28°C, 200 rpm. The citric acid detection kit was used to detect the citric acid content in the two cultures for 24 h. **(A)** Fecal 16S sequencing results and serum metabolomics for joint analysis. **(B)** Detection of the content of CA in the culture of *Massilia* by CA kit (*n* = 3, means ± SEM, Student's *t*-test, * *P* < 0.05, ** *P* < 0.01).

Based on the above results, we speculated that citric acid production might be metabolized by intestinal microbes. Combined analysis of fecal 16S sequencing results and serum metabolomics showed a positive correlation of citric acid with intestinal *Massilia* (Figure 7A). In order to verify whether *Massilia* produced citric acid, it was cultured in R2A liquid medium for 24 h *in vitro*, and the content of citric acid in the medium was detected and compared with the pure R2A liquid medium. We observed that compared with the control group, the citric acid content in the culture medium of the *Massilia* group was almost doubled (Figure 7B).

Therefore, we could show that *H. illucens* larvae feed increased the content of *Massilia* in the intestine, whose metabolite—citric acid—improved the poor immune performance and intestinal barrier damage of piglets caused due to ETEC K88 infection.

## Discussion

ETEC K88 strain is a typical strain that causes post-weaning diarrhea in piglets. ETEC infection can cause poor immune performance and intestinal barrier damage in piglets. The addition of oxytetracycline and other feed antibiotics that inhibit ETEC in the diet significantly reduced the diarrhea of weaned piglets and improved the growth performance of piglets to a certain extent (23). However, due to the total ban on the use of growth-promoting drug feed additives, it is urgent to develop functional and safe feed additives that can replace antibiotics. The larvae of *H. illucens*, which have been widely used in animal feed, such as broilers, laying hens, and weaned piglets have abundant antimicrobial peptides. Previous studies have shown a positive effect of the addition of 4 and 8% *H. illucens* larvae to the weaned piglet's diet in terms of the cecal microbiota and small intestinal mucin dynamics of the piglet (8). The results further confirmed the utility of adding *H. illucens* larvae powder in pig diets. In addition, the addition of *H. illucens* larvae in the diet stimulated the non-specific immune response of broilers and improved their survivability unde*r Salmonella gallinarum* infection (24). However, the specific effect of *H. illucens* larvae on ETEC-infected piglets is currently unclear.

Previous studies have shown that promoting the secretion of intestinal IgA is effective against the colonization and translocation of ETEC in the intestine (22). At the same time, natural IgG is used as a feed supplement for the production of modern pigs, which can reduce post-weaning diarrhea (PWD) and prevent and treat other intestinal infectious diseases (25). We found that after ETEC infection, the content of IgA and IgG in the serum of the piglets in the 4% *H. illucens* larvae + K88 and 8% *H. illucens* larvae + K88 groups increased gradually, which correlated positively with the dosage of *H. illucens* larvae. Therefore, it is speculated that *H. illucens* larvae may counter the challenge of ETEC by increasing the level of immunoglobulin in piglets. A series of fine-regulated events cause B cells to mature and differentiate into antibody-secreting plasma cells (26). During the analysis of the serum metabolites of weaned piglets, gradually increasing citric acid in the serum of *H. illucens* feed piglets was a noteworthy observation among the differential metabolites. Previous studies have shown that adding citric acid to broiler feed can significantly improve the immune status of its immunocompetent cells. Citric acid can be converted into acetyl-CoA in the cytoplasm for fatty acid synthesis (27), which is necessary for B cell differentiation (28). Therefore, the process of B cell maturation and differentiation into plasma cells that secrete IgA and IgG may involve citric acid. To verify this, citric acid was given to mice as pre-protection before ETEC infection. Then, immunoglobulin isotype analysis in the mouse serum showed significantly increased levels of IgG and IgA secretion in the serum of the CA pre-protection group compared with the ETEC group, and the CA group alone had the same effect. An increase in IgA and IgG levels may be due to the stimulation of citric acid leading to B cell proliferation or differentiation.

When the inactivated B cells are stimulated by an antigen *via* IgM, these cells undergo Ig class switching recombination (CSR) to produce antibodies of different isotypes but having the same antigen specificity as IgM, such as IgA and IgG (29). Many cells provide the costimulation and cytokine signals required by IgCSR and B cells to produce antibodies (29). Macrophages are known to play a role in response to antigen presentation to B cells and T cells and in the secretion of BAFF and pro-inflammatory (i.e., IL-6, TNF-α) and anti-inflammatory (i.e., TGF-β, IL-10) cytokines. Our study first confirmed that upon LPS stimulation of RAW264.7 cells, the CA pretreatment group had enhanced expression of BAFF and IL-10 in cells and in the culture medium and reduced expression of TGF-β. IL-10 had been shown to promote Ig conversion to produce IgA (30). Therefore, our data show that citric acid stimulated the production of IgA and IgG by promoting the secretion of cytokines related to B cell differentiation by RAW264.7 cells. Citric acid also inhibited the excess release of pro-inflammatory cytokines IL-6, TNF-α, and IL-1β by RAW264.7 cells under LPS injury.

ETEC K88 colonization in the host intestine, which damages the intestinal barrier function and triggers an inflammatory response (31). The addition of *H. illucens* larvae (especially the 4% supplement group) down-regulated the expression of the gene coding for pro-inflammatory cytokine IFN-γ in the colonic mucosa of fattening pigs and simultaneously up-regulated the anti-inflammatory cytokine IL-10 expression (13). We found that *H. illucens* played a similar role in the ileum of weaned piglets. Interestingly, *H. illucens* significantly attenuated the ileal inflammation caused by ETEC infection of piglets. The intestinal mucosal barrier is an integral part of maintaining the homeostasis of the intestinal microenvironment, of which the intestinal epithelial cell is an important part, and the intestinal tight junction adjacent to a single cell serves as another physical structure. This tight junction complex comprises about 50 proteins that are basically categorized into structural and functional units (32, 33). The key role of tight junctions is to close the gaps between epithelial cells, thereby limiting the infiltration of microorganisms or other antigens into the circulation (34). In early-weaned piglets, ETEC K88 infection can increase the permeability of tight junctions (35), and our results were consistent with this report. *H. illucens* larvae feed can up-regulate the expression of intestinal barrier-forming genes, such as ZO-1, occludin, and mucin-1 in the colon mucosa of fattening pigs (13) and has the same effect in the ileum of weaned piglets. The larvae of *H. illucens* promoted the expression of ZO-1 in the ileum of piglets with ETEC infection, indicating that it possibly enhances the integrity of the mucus layer and produces a host-friendly intestinal environment that can resist pathogen infection.

The relationship between apoptosis and the inflammatory response has been studied in recent years. For example, abnormal apoptosis is observed in the pathogenesis of inflammatory bowel disease and is affected by many factors. The pathogenesis of inflammatory bowel disease occurs in the form of apoptosis (36); it is an active process involving the activation, expression, and regulation of a series of genes. Among the genes involved in regulating mammalian cell apoptosis, members of the Bcl-2 family are functionally classified as anti-apoptotic or pro-apoptotic, and they regulate cell apoptosis by inducing or inhibiting cell death (37). Bcl-2 and Bax are members of the Bcl-2 family of proteins. Bcl-2 has an anti-apoptotic effect and has been shown to promote cell survival in many systems (38), while Bcl-2-related protein × (Bax) is pro-apoptotic and is the main inhibitor of Bcl-2 (39). Studies in mammals have shown that changes in the Bax to Bcl-2 expression ratio in cells affect the release of mitochondrial cytochrome c (40), and an increase in this ratio can induce apoptosis (41). Thus, adding *H. illucens* larvae feed inhibited the apoptosis of piglet ileal cells caused by ETEC and protected the intestinal barrier from damage.

LPS induces IPEC-J2 cells to overproduce pro-inflammatory factors IL-6, TNF-α, and IL-8, causing inflammation (42). Consistent with this, we found that compared with the LPS group, the CA pre-protection group had significantly reduced levels of pro-inflammatory factors and enhanced expression of tight junction proteins. At the same time, there was an increase in the ratio of Bcl-2/Bax, indicating that citric acid protected IPI-2I cells from LPS-induced damage. Therefore, our results firmly indicate that citric acid protects the intestinal barrier against pathogen-induced epithelial damage both *in vivo* and *in vitro*.

The above research results proved that citric acid derived from *H. illucens* supplement improved the immune performance of piglets and protected their intestinal health. The use of larvae of *H. illucens* as a feed ingredient has been reported to affect the intestinal microbiota and microbial metabolites of laying hens or broilers (24). Therefore, we speculated that citric acid content in serum might be due to intestinal microorganisms fermentation. In this study, *Massilla* should be highlighted. *Massilia* strains belong to the Bacteria, Proteobacteria, Betaproteobacteria, Burkholderiales, Oxalobacteraceae, gram-negative bacteria (43). The members of the genus *Massilia* show a variety of physiological activities, such as *Ma*ssilia umbonata LP01T, a new species able to accumulate poly-β-hydroxybutyrate (44). *Massilia* spp. have been confirmed as the pathogenic bacteria causing otitis media (45). Due to the wide distribution, strong adaptability to the environment, and potentially important application value of *Massilia* strains, their application has always been a focus of research. The studies have mainly been concentrated on enzyme production and soil remediation. However, there are few reports on *Massilia* applications in animal feeding. In this study, we proved that *Massilia* spp. have the potential to produce CA, which contributes to the maintaining of mucus integrity, activating B cells to mature plasma cells, promoting IgA, IgG secretion restricting pathogen colonization in piglets. Our findings not only confirm the advantages of *H. illucens* in animal feed but also depict the mechanisms mediated by CA. Furthermore, the potential use of *Massilia* in microecological modulation should be highlighted. Nevertheless, further rigorous verification and evaluation are still needed.

## Conclusion

**Figure 8:**
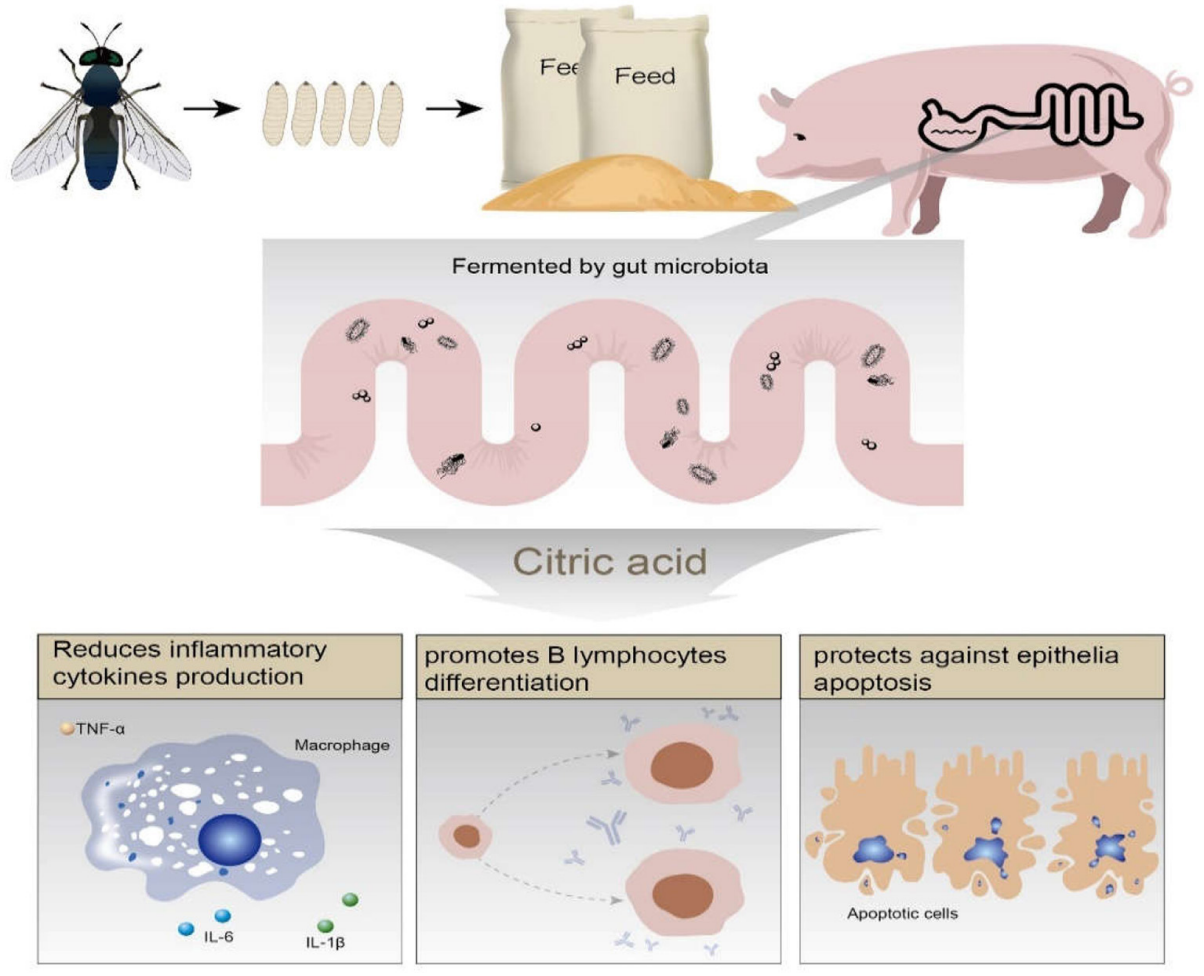
Schematic representation of the proposed mechanism.

The protective effects of *Hermetia illucens* feed in ETEC induced piglets diarrhea possibly mediated by microbiota fermented citric acid, which promoted B lymphocyte differentiation and presented anti-apoptotic property on epithelial cells (Figure 8).

## Data Availability Statement

The datasets presented in this study can be found in online repositories. The names of the repository/repositories and accession number(s) can be found at: NCBI: PRJNA750611.

## Ethics Statement

The animal study was reviewed and approved by Institutional Animal Care and Use Committee of Zhku (Permit Number: 202002007). Written informed consent was obtained from the owners for the participation of their animals in this study.

## Author Contributions

WW, YL, and YH designed the research and have primary responsibility for final content. WW, ML, BY, and XJ conducted the research. ML and BY: model building and sample extraction. ML, XJ, and HX: RNA-seq. MZ, ZW, and GX: molecular mechanism including RT-PCR and western blot. XZ and HX analyzed the data. WW, BL, and ZX wrote the paper. All authors contributed to the article and approved the submitted version.

## Funding

This work was supported by the National Natural Science Foundation of China (31872442) and Innovative Team Projects of Ordinary Colleges and Universities in Guangdong Province (2020KCXTD019).

## Conflict of Interest

The authors declare that the research was conducted in the absence of any commercial or financial relationships that could be construed as a potential conflict of interest.

## Publisher's Note

All claims expressed in this article are solely those of the authors and do not necessarily represent those of their affiliated organizations, or those of the publisher, the editors and the reviewers. Any product that may be evaluated in this article, or claim that may be made by its manufacturer, is not guaranteed or endorsed by the publisher.
